# Degree of SGLT1 phosphorylation is associated with but does not determine segment‐specific glucose transport features in the porcine small intestines

**DOI:** 10.14814/phy2.13562

**Published:** 2018-01-15

**Authors:** Stefanie Klinger, Patrick Lange, Elisabeth Brandt, Karin Hustedt, Bernd Schröder, Gerhard Breves, Jens Herrmann

**Affiliations:** ^1^ Department of Physiology University of Veterinary Medicine Hannover, Foundation Hannover Germany

**Keywords:** GLUT2, intestinal axis, intestinal glucose transport, SGLT1, Ussing chamber

## Abstract

Glucose‐induced electrogenic ion transport is higher in the porcine ileum compared with the jejunum despite equal apical abundance of SGLT1. The objective of this study was a detailed determination of SGLT1 and GLUT2 expressions at mRNA and protein levels along the porcine small intestinal axis. Phosphorylation of SGLT1 at serine 418 was assessed as a potential modulator of activity. Porcine intestinal tissues taken along the intestinal axis 1 h or 3 h after feeding were analyzed for relative mRNA (RT‐PCR) and protein levels (immunoblot) of SGLT1, pSGLT1, GLUT2, (p)AMPK, β_2_‐receptor, and PKA substrates. Functional studies on electrogenic glucose transport were done (Ussing chambers: short circuit currents (*I*_sc_)). Additionally, effects of epinephrine (Epi) administration on segment‐specific glucose transport and pSGLT1 content were examined. SGLT1 and GLUT2 expression was similar throughout the small intestines but lower in the duodenum and distal ileum. pSGLT1 abundance was significantly lower in the ileum compared with the jejunum associated with significantly higher glucose‐induced *I*_sc_. SGLT1 phosphorylation was not inducible by Epi. Epi treatment decreased glucose‐induced *I*_sc_ and glucose flux rates in the jejunum but increased basal *I*_sc_ in the ileum. Epi‐induced PKA activation was detectable in jejunal tissue. These results may indicate that SGLT1 phosphorylation at Ser418 represents a structural change to compensate for certain conditions that may decrease glucose transport (unfavorable driving forces/changed apical membrane potential) rather than being the cause for the overall differences in glucose transport characteristics between the jejunum and ileum.

## Introduction

The small intestines of monogastric animals are the major sites for absorption of nutrients such as fatty acids, amino acids, and monosaccharides. Among the latter, glucose represents the most abundant molecule. According to the classical model, glucose uptake from the intestinal lumen into the enterocytes is mediated by the Na^+^/glucose co‐transporter 1 (SGLT1) as a secondary active, electrogenic transport system, while intracellularly accumulated glucose is released by facilitated diffusion *via* the basolateral located glucose transporter 2 (GLUT2) (Wright et al. 2007). For completeness, two complementary models have to be mentioned which may be of physiological relevance at high luminal glucose concentrations. Kellett and colleagues proposed an apical insertion of GLUT2 when SGLT1 becomes saturated (Kellett and Helliwell 2000). According to the Pappenheimer hypothesis, solvent drag driven paracellular glucose transport exceeds transcellular transport at high glucose availability (Pappenheimer and Reiss 1987).

As shown in mice, the gene expression of SGLT1 or GLUT2 changes along the small intestines with highest levels in the proximal segments (Yoshikawa et al. 2011). SGLT1 protein content in rat small intestines was highest in the jejunum compared with the duodenum or ileum (Balen et al. 2008). It has also been shown that at least duodenal expression levels of SGLT1 and GLUT transporters vary between humans and rodents indicating species‐related distribution patterns of glucose transporters along the intestinal axis (Kim et al. 2007). However, equivalent human data regarding glucose transporter abundance along the gastrointestinal tract are rare although there is a rising incidence of metabolic disorders based on disturbed glucose absorption in Western societies. Increases in Na^+^‐dependent glucose transport as well as in the abundance of monosaccharide transporters including SGLT1 in the duodenum of diabetic human patients have been described (Dyer et al. 2002). However, in this previous study, differences between intestinal segments were not investigated.

Omnivore living pigs can be considered to subsist on equal diets compared with humans. The anatomy of the gastrointestinal tract, its physiology, and absorptive processes are much more in agreement in humans and pigs compared with the well‐established rodent animal models (Nejdfors et al. 2000; Deglaire and Moughan 2012; Varum et al. 2012). Nevertheless, detailed information about intestinal distribution or activity of glucose transporters in pigs is rare. Expression of SGLT1 and GLUT2 mRNA in the porcine jejunum had been shown earlier (Aschenbach et al. 2009). Another study examined the influence of long‐term regulation by low versus high carbohydrate diets during weaning of piglets on intestinal glucose uptake and SGLT1 mRNA expression in proximal, mid, and distal small intestine (Moran et al. 2010). The authors found that the mid small intestines were most reactive to high carbohydrate diet resulting in increased SGLT1 mRNA expression and glucose uptake; at lower dietary carbohydrate levels, glucose uptake was similar in all three segments and SGLT1 mRNA abundance was less divergent compared with the high carbohydrate diet.

We have shown previously that the electrogenic glucose transport in the small intestines of growing pigs is significantly higher in the distal part compared with the middle segment, while the apical abundance of SGLT1 protein is similar (Herrmann et al. 2012). The discrepancy between protein abundance and transport rate might have been induced by different degrees of SGLT1 phosphorylation as it has been reported that regulation of electrogenic glucose transport in *Xenopus laevis* oocytes‐expressing rabbit, human or rat SGLT1 largely depends on PKC‐ or PKA‐mediated SGLT1 integration into the membrane (Hirsch et al. 1996; Wright et al. 1997). However, activation of PKA also mediated direct phosphorylation of overexpressed SGLT1 at serine 418 (Ser418) in Chinese hamster ovary cells resulting in an increase in transporter affinity to glucose by conformational changes (Subramanian et al. 2009). Similar observations were reported using small intestinal loops from rats perfused with epinephrine (Epi) or norepinephrine (Ishikawa et al. 1997). The catecholamines could stimulate glucose transport by increasing SGLT1 apical abundance as well as glucose‐binding ability. The study revealed PKA‐mediated SGLT1 phosphorylation after binding of Epi to β‐adrenergic receptors. However, no data are available concerning effects of PKA on SGLT1 and electrogenic glucose transport in the porcine small intestines.

Therefore, in order to clarify the physiological basis for segmental diversity in glucose absorption in the porcine small intestines, this study aimed at investigating (1) glucose transporter expression along the porcine small intestinal axis including basal phosphorylation status of SGLT1 at Ser418 in relation to progression of digestion, (2) influence of Epi on electrogenic glucose transport in the porcine mid jejunum as well as ileum, and (3) effects of Epi on PKA activity and SGLT1 Ser418 phosphorylation status.

## Materials and Methods

### Animals and tissue sampling

In total, 35 female growing pigs *(Sus scrofa domestica)* of a conventional crossbreed *(German Landrace x Large White)* aging 10–12 weeks were housed in groups of four for at least 1 week. Please note that the experiments described below were not carried out with all animals. The number of animals used for each experiment or correlation is always indicated. The pigs were fed twice daily with a conventional fattening diet (13.4% MJ ME kg^‐1^) and had access to drinking water ad libitum. All animals received treatment and care according to the German Animal Protection Act which complies with the German Research Council's criteria and the EC Directive 2010/63/EU for animal experiments.

The pigs were slaughtered by stunning with subsequent carotid artery bleeding. To examine an effect between the last feed intake and slaughtering on jejunal and ileal glucose and starch contents, the animals were sacrificed either 1 h (“1 h‐group”, *N* = 28) or 3 h (“3 h‐group”, *N* = 7) after morning feeding. These time intervals between feed intake and slaughtering should result in different starch and glucose contents along the intestinal axis as induced by kinetics of stomach emptying. Some data on digesta flow at the duodenum indicate low flow rates in the first hour after feeding and a maximum after 3 h (Weisthoff 1990), while other data suggest the maximum flow rate to occur earlier and to be dependent on the feeding regime and diet (Freeman et al. 1968; Braude et al. 1976; Krawielitzki et al. 1990; Weisthoff 1990). For this reason, chyme was analyzed for glucose and starch contents, especially because no data are available for the jejunal segment which was used in this study. For the ileal digesta flow, a minimum around feeding and a maximum after 3–4 h after feeding was shown (Jørgensen et al. 1997).

**Figure 1:**
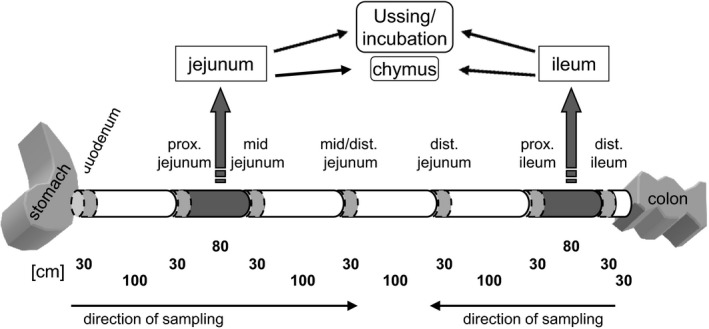
Scheme of tissue sampling. Samples of 30 cm length were taken from seven defined locations along the small intestinal axis for RNA and protein preparations (bright gray). Two intestinal segments of 80 cm length were taken for Ussing chamber and Epi incubation studies (dark gray). The chyme in these intestinal segments was collected for starch and glucose determinations. The samples from duodenum to mid/distal jejunum were taken beginning from the pylorus, while the samples from distal ileum to distal jejunum were taken beginning from the ileocaecal valve as indicated by arrows.

The mean body weights at slaughtering were 25.0 ± 2.8 kg and 25.2 ± 2.1 kg in the 1 h‐ and 3 h‐group. Immediately after slaughtering, tissues were taken from the segment between 1.6 and 2.4 m distal to the pylorus, defined as jejunum, and from the segment between 0.6 and 1.4 m proximal to the ileocaecal valve, defined as ileum, for electrophysiological and transport studies in Ussing chambers and Epi incubation procedures. Chyme of both intestinal segments was collected and stored at −20°C for analyses of starch and glucose contents. Muscle‐stripped mucosal tissue samples were taken from seven locations along the small intestinal axis (Fig. 1), shock‐frozen in liquid nitrogen and stored at −80°C for analyses of glucose transporter abundance.

### Ussing chamber technique – overview and methodical considerations

Ussing chamber studies were performed with muscle‐stripped mucosal tissues from the jejunum and ileum, thereby paying attention not to use lymphatic tissue. Twelve Ussing chambers (K. Mussler, Aachen, Germany) per intestinal segment and animal were used. Immediately after sampling and collection of chyme, the segments were rinsed with ice‐cold saline (0.9% NaCl (w/v)) and kept in a modified Krebs‐Henseleit buffer (continuously aerated with carbogen) until mounting in Ussing chambers with an exposed surface of 1.13 cm^2^(Breves et al. 2000; Herrmann et al. 2012). Epithelia were mounted within 45 min after stunning.

As described in detail in the following subsections, both, electrophysiological measurements were done as well as the determination of unidirectional mucosal‐to‐serosal flux rates (*J*_ms_) and it will shortly be discussed, which method was used for which issue and for what reason.

Since short circuit currents (*I*_sc_) are a measure for the transepithelial net ion transfer, changes in *I*_sc_ do not necessarily reflect changes in transepithelial Na^+^ transfer after glucose administration due to other ion movements. To account for this, *J*_ms_ after the addition of glucose were measured exemplarily for eight animals in previous experiments. When analyzing mean *J*_ms_ (*I*_sc_: jejunum 2.9 ± 1.4 *μ*E cm^−2^ h^−1^ vs. ileum 7.5 ± 2.0 *μ*E cm^−2^ h^−1^, *P *<* *0.001; *J*_ms_: jejunum 1324 ± 771 nmol cm^−2^ h^−1^ vs. ileum 2296 ± 518 nmol cm^−2^ h^−1^, *P *<* *0.01), the difference in ∆*I*_sc_ between jejunal and ileal tissues (Herrmann et al. 2012) could also be detected. From these data, one may calculate the proportion of Δ*I*_sc_ that is not caused by Na^+^ movement. Regarding the means it may appear as if all of the Δ*I*_sc_ in jejunal samples is due to Na^+^ movement, whereas ileal Δ*I*_sc_ is higher than it could be explained by the flux data. This might have been mediated by Ca^2+^‐dependent Cl^−^ secretion (Yin et al. 2017), but having a closer look on the jejunal data, there is also a correlation between Δ*I*_sc_ and the proportion of Δ*I*_sc_ that could not be explained by Na^+^ movement (data not shown). It has to be clarified in general and for the porcine small intestines which ion transport processes are the cause for this discrepancy between Δ*I*_sc_ and *J*_ms_. To account for this and to account for the fact that Epi is able to influence the conductance for several ions via activating PKA, *J*_ms_ was measured to assess effects of Epi on glucose transport. Only electrophysiological measurements were performed in this study which examines differences between jejunal and ileal tissues with regard to the time of feeding.

Another remarkable point is the mucosal glucose concentration used in this study. 5 mmol L^−1^ glucose were used in order to ensure, that SGLT1 is the sole acting transport system. Results from Kellett and colleagues suggest that the apical amount of GLUT2 they detected did not change between 0 and 10 mmol L^−1^ glucose (Kellett and Helliwell 2000) although no quantification is given in this paper. Paracellular glucose transport should not be relevant at a mucosal glucose concentration of 5 mmol L^−1^ (Atisook et al. 2002).

### Ussing chamber technique – electrophysiological measurements

For electrophysiological examinations, the epithelia were equilibrated for 30 min in a serosal buffer containing 5 mmol L^−1^ glucose and a mucosal buffer without glucose. Both buffer solutions were based on (mmol L^−1^) 113.6 NaCl, 5.4 KCl, 0.2 HCl, 1.2 MgCl_2_, 1.2 CaCl_2_, 21.0 NaHCO_3_, 1.5 Na_2_HPO_4_, 1.2 mannitol, and 0.01 indomethacin. Further ingredients in the serosal buffer were (mmol L^−1^) 7.0 HEPES and 6.0 Na‐gluconate, while the mucosal buffer contained (mmol L^−1^) 20.0 HEPES and 6.0 NaOH. Both buffers had an osmolality of 293 mosmol kg^−1^ and a pH of 7.4 at 37°C.

Subsequently, glucose concentration in the mucosal buffer was adjusted to 5 mmol L^−1^ and to assure osmotic stability 5 mmol L^−1^ mannitol was added to the serosal buffer. *I*_sc_ was monitored continuously and glucose‐induced responses were expressed as the differences (∆*I*_sc_) between initial and maximal *I*_sc_ in between 10 min after application. 50 *μ*mol L^−1^ (‐)‐Epinephrine (Sigma, Taufkirchen, Germany) or 0.5 N HCl as solvent control were added to serosal buffer 90 min after glucose administration. For further analyses serosal administration of 50 *μ*mol L^−1^ of the β‐adrenoceptor inhibitor propranolol (Sigma) or serosal and mucosal addition of 10 *μ*mol L^−1^, respectively, of the PKA inhibitor H‐89 (Sigma) were done 15 min prior to Epi application. Epithelial integrity was controlled continuously in all Ussing chambers by measuring tissue conductance (G_t_) using Ohm's law.

### Ussing chamber technique – flux rate measurements

Determination of glucose flux rates was carried out by adding 133.2 kBq [^3^H]‐glucose (Hartmann Analytic GmbH, Brunswick, Germany) to the mucosal buffer together with 5 mmol L^−1^ unlabeled glucose. After 30‐min incubation, 500 *μ*L samples were collected at 15‐min intervals from the unlabeled serosal buffer compartment and the buffer volume was filled up. Solvent or 50 *μ*mol L^−1^ Epi were added to the serosal buffer after the fourth sampling. The incubations were stopped after a further four samplings. In parallel to the first and the last serosal sample collections, 50 *μ*L of labeled mucosal buffer were taken and filled up to 500 *μ*L with unlabeled buffer for flux rate calculations.

All samples were mixed with 4.3 mL scintillation liquid (Rotiszint Eco‐Plus, Carl Roth GmbH, Karlsruhe, Germany) for determining specific activity (decays per minute, dpm) in a 2500 TR Tri‐Carb liquid scintillation analyser (Packard Instrument Company, Downers Grove, IL). Unidirectional glucose mucosal‐to‐serosal flux rates (*J*_ms_) were calculated according to standard equations (Schultz and Zalusky 1964). Δ*J*_ms_ (nmol cm^−2^ h^−1^) was calculated between the flux periods of 15 minutes for control and Epi‐treated chambers.

### Incubation of intestinal tissue

For investigation of Epi effects on the apical abundance of total SGLT1 or phosphorylated SGLT1 at Ser418 (pSGLT1) 1.5 cm^2^ stripped mucosa taken from the jejunum and ileum were incubated in parallel to Ussing chamber studies in 15 mL continuously aerated serosal buffer solution. After 20 min of equilibration the tissue samples were either incubated with 50 *μ*mol L^−1^ (‐)‐epinephrine or solvent (0.5 N HCl) for 15 min. In parallel, samples were treated with 20 *μ*mol L^−1^ propranolol or solvent 20 min prior to Epi administration. Subsequently, the tissue samples were shock‐frozen in liquid N_2_ and stored at −80°C until use.

### Analyses of intestinal starch and glucose contents

Chyme samples were thawed, weighed, and freeze‐dried. One gram of dried chyme was dissolved (60°C in 5 mL 8 mol L^−1^ HCl and 20 mL DMSO, 30 min; H_2_O ad 100 mL, pH 4‐5 was titrated with 5 mol L^−1^ NaOH). Measurements of starch and glucose contents were performed in duplicate using a photometric UV‐test kit according to the manufacturer's manual (r‐biopharm, Darmstadt, Germany).

### RNA isolation and quantitative RT‐PCR

Total RNA was isolated from stripped intestinal mucosa using the RNeasy Plus Mini‐Kit (Qiagen, Hilden, Germany) according to the manufacturer's manual and cDNA was generated from 200 ng RNA with the TaqMan^®^‐Reverse Transcription Reagents Kit (Applied Biosystems, Darmstadt, Germany).

GLUT2 gene expression (gene name: *SLC2A2*; Acc. No. NM_001097417; sense primer: 5′‐TAGAGAAGGCAGGGCGAC‐3′; antisense primer: 5′‐CATCCAAGGCAATTTATCCAGTA‐3′; 110 bp fragment) was determined by quantitative RT‐PCR using the SYBR Green^®^ assay as described elsewhere (Wilkens et al. 2016). Expression of SGLT1 (gene name: *SLC5A1*; Acc. No.: NM_001164021; sense primer: 5′‐GCTTTGAATGGAATGCTCTGATT‐3′; antisense primer: 5′‐GCATCGTCACCACCCCTG‐3′; 87 bp fragment; probe: 5′ FAM‐AATGGGGACAAACAGCCAGCC‐BBQ 3′ 3′) and as endogenous reference ribosomal protein, RPS18 (Acc. No.: NM_213940.1; sense primer: 5′‐TGCTATCACTGCGATTAAGGTGTA‐3′; antisense primer: 5′‐GCATAATGGTGATTACACGTTCCA‐3′; 131 bp fragment; probe: 5′ FAM‐ ATCGACCTCACCAAGAGGGCAGG‐BBQ 3′) was quantified by TaqMan^®^ assays as described previously in detail (Kunert‐Keil et al. 2006). The primers and probes were obtained from TIB MOLBIO (Berlin, Germany) and the quantitative RT‐PCRs were performed in duplicate with the thermocycler CFX96TM (Bio‐Rad Laboratories GmbH, Munich, Germany). To generate calibration curves for determining of absolute copy numbers, DNA fragments produced by the primers for detecting of GLUT2, SGLT1 and RPS18 were cloned into the pGEM‐T easy vector (Promega, Mannheim, Germany) and sequenced (GATC Biotech, Konstanz, Germany). Sequences were verified using the NCBI Blast software (http://blast.ncbi.nlm.nih.gov/Blast.cgi). Copy numbers of RSP18 were stable along the intestinal axis in both groups but copy numbers were slightly higher in the 3 h‐group.

### Tissue preparations and immunoblotting – overview

To apply immunoblotting for detecting nutrient transporters, the *β*‐receptor and intracellular regulatory proteins, different methods of tissue preparation were used to enrich the subcellular fractions to which each protein is located to. Table 1 presents a detailed overview of which preparation techniques were used to detect the different proteins and how the respective antibodies were used. The experimental procedure for tissue preparation steps and immunoblotting is described below. Briefly, brush border membranes (BBM) were used for detecting SGLT1, while crude membrane preparations (containing both the apical and the basolateral membranes) were used for detecting GLUT2 and *β*‐receptor.

**Table 1:** Description of antibodies and western blot conditions

	Prep	Protein loading	Denaturation	Blocking (90 min, RT)	PAK (overnight 4°C)	SAK (90 min, RT)
SGLT1	BBM	12 *μ*g	40°C, 15 min	5% MP/TBST	1:2000	1:20,000
pSGLT1	BBM	12 *μ*g	40°C, 15 min	5% MP/TBST	1:2000	1:15,000
GLUT2	CM	20 *μ*g	70°C, 10 min	2,5% MP/TBST	1:2000	1:30,000
β_2_‐receptor	CM	12 *μ*g	70°C,10 min	5% MP/PBST	1:200	1:10,000
PKA substrate	Cyto	9 *μ*g	95°C, 5 min	5% MP/TBST	1:1000*	1:2000
AMPK	Cyto	9 *μ*g	95°C, 5 min	5% MP/TBST	1:1000*	1:2000
pAMPK	Cyto	9 *μ*g	95°C, 5 min	5% MP/TBST	1:1000*	1:2000

Antibody‐specific parameters are indicated for used preparations (Prep) (BBM, brush border membrane; CM, crude membrane; cyto, cytosol) protein loading, denaturation conditions, blocking conditions (MP/TBST, MP/PBST = dry milk in TRIS or phosphate‐buffered saline with 0.1% Tween 20) and incubation times as well as dilutions of primary (PAK) or secondary antibodies (SAK). Antibodies were diluted in the respective blocking solution with exception of PAKs indicated by “*” which were diluted in 5% bovine serum albumin/TBST. pSGLT1 was detected using SuperSignal^®^ West Femto Maximum Sensitivity Substrate instead of SuperSignal^®^ West Dura Extended Duration Substrate (both Thermo Scientific).

In order to test whether the applied experimental conditions do in fact lead to an activation of PKA, two assays were performed. We used a nonradioactive commercial assay (see below). For this purpose, whole cell lysates were prepared according to the instruction manual. We also used an antibody directed against the phosphorylated PKA consensus sequence (Table 1) allowing the detection of all proteins in a sample that are phosphorylated at this sequence. Instead of a single western blot band, the whole lane intensity was measured and normalized on total protein per lane as determined after Indian Ink staining (see below).

Since estimating the relative amount of phosphorylated PKA substrates is rather an indirect and rough measure of PKA activity, we additionally measured the phosphorylation level of AMPK as an indirect downstream target of PKA. A direct PKA downstream kinase is LKB1. Phosphorylation of AMPK by LKB1 at threonine 172 (Thr172) reflects its activation (Carling et al. 2008) and was used to assess Epi effects on its activity. PKA phosphorylated substrates as well as AMPK/pAMPK were detected using cytosol preparations.

### Preparation of BBM, crude membranes, and cytosol

BBM were prepared by CaCl_2_ precipitation from stripped intestinal mucosa. Frozen tissue samples were thawed in ice‐cold buffer solution (2 mmol L^−1^ Tris base, 50 mmol L^−1^ mannitol, 0.1 mmol L^−1^ PMSF; pH 7.1) and homogenized with an Elvehjem‐Potter (Janke & Kunkel, Staufen im Breisgau, Germany). After drop wise addition of CaCl_2_ under stirring (10 mmol L^−1^ final) and 30 min incubation on ice, the suspension was centrifuged (2000*g*, 20 min, 4°C). The supernatant was centrifuged (25,000*g*, 30 min, 4°C) and the pellet was resolved in buffer (10 mmol L^−1^ Tris base, 150 mmol L^−1^ NaCl; pH 7.4) containing protease and phosphatase inhibitor cocktails (Sigma) and stored at −20°C. Cytosol was obtained after homogenization in buffer (10 mmol L^−1^ Tris, 0.5 mmol L^−1^ EDTA, 0.1% SDS, pH 7.4) and centrifugation (17,500*g*, 30 min, 4°C). The CM preparations were performed as described previously (Herrmann et al. 2012). Protein contents were measured by the Bradford method (Bradford 1976) using the Bio‐Rad Protein Assay (Bio‐Rad Laboratories).

### SDS‐PAGE and immunoblotting

Samples were solubilized in Laemmli buffer and denatured (Table 1). Resulting protein extracts were resolved on 8.5% (w/v) Laemmli gels in SDS‐polyacrylamide gel electrophoresis (SDS‐PAGE) under reducing conditions in Tris‐glycine buffer. The proteins were electroblotted onto Protran™ nitrocellulose membranes (Amersham, Freiburg, Germany) which were subsequently blocked and incubated with the respective primary antibodies (overnight, 4**°**C).

The following polyclonal rabbit primary antibodies were used according to Table 1: SGLT1 (ab14686, Abcam, Cambridge, United Kingdom), pSGLT1 (epitope: KIRKRApSEKELMI, custom made by Perbio Science, Bonn, Germany), GLUT2 (ABIN310208, Antibodies‐online, Aachen, Germany), *β*_2_‐receptor (ab71219, Abcam; 1:200), Phospho‐(Ser/Thr) PKA Substrate Antibody (#9621, Cell Signaling Technology, Danvers), AMPK (#5832, Cell Signaling Technology), and pAMPK (#2535, Cell Signaling Technology). Immunocomplexes were detected with horseradish peroxidase‐conjugated goat anti‐rabbit antibody (A9169, Sigma‐Aldrich). Chemiluminescence signals were detected using SuperSignal^®^ West Dura Extended Duration Substrate or SuperSignal^®^ West Femto Maximum Sensitivity Substrate (Thermo Scientific) according to the manufacturer's manuals and the ChemiDoc™ MP imaging system. Densitometric analysis of detected bands was performed with the Image Lab 5.2 software (Bio‐Rad Laboratories). Due to comparison of different intestinal segments with unknown segment depending expression levels of classical housekeeping genes like *β*‐actin or GAPDH, normalization of protein expression data occurred by densitometric analysis of the total protein loading of the respective gel lane (documented by Indian ink staining). For this, the membranes were incubated over night with fountain pen ink (4001, Pelikan, Hannover, Germany) including 2% (v/v) glacial acetic acid. Subsequently, the membranes were washed with distilled water. After drying, the band intensities were detected by the ChemiDoc™ MP imaging system.

For an estimation of pSGLT1 contents relative to total SGLT1, the standard protocol was modified. After pSGLT1 detection, the membrane was incubated with a pH 2.0 stripping buffer (0.2 mol/L Glycin, 0.05% Tween 20, 1% SDS, 45 min, RT). The stripped membrane was washed twice with TBST and blocked again. Afterward the membrane was incubated with the SGLT1‐specific antibody (Table 1).

Abundance of adrenergic β_2_‐receptor or pSGLT1 (Ser418) in the jejunum or ileum was estimated for each animal by determining the relative abundance in the respective intestinal samples surrounding the segments used for Ussing chamber examinations and subsequent calculation of the respective mean value for each segment.

### Detection of PKA activity

In order to figure out Epi‐induced changes in the activity of PKA, the PepTag^®^ Assay for Non‐Radioactive Detection of cAMP‐Dependent Protein Kinase (Promega) was used according to the manufacturer's instructions. PKA‐mediated phosphorylation of a fluorescence labeled target peptide resulted in changes of the peptide's net charge which could be detected using 0.8% agarose gel electrophoresis and visualization at 340 nm using the ChemiDoc™ MP imaging system (Bio‐Rad Laboratories). PKA activity was calculated by densitometric analysis of resulting band intensities of phosphorylated and non‐phosphorylated peptides by the Image Lab 5.2 software (Bio‐Rad Laboratories).

### Statistics

The data are presented as arithmetic means ± SD. Animal numbers (N) are indicated. Statistical analyses of changes in *I*_sc_ or *J*_ms_, of intestinal starch or glucose contents and *β*_2_‐receptor abundance were performed using unpaired Student's *t*‐test, while Epi effects on SGLT1 phosphorylation (Ser418) or PKA activity were analyzed with paired Student's *t*‐test. A multiple *t*‐test was used for the data regarding Epi effects on m/s glucose flux rates. Expression data along the intestinal axis were compared by one‐way ANOVA using the Kruskal–Wallis test with Dunn′s multiple comparison post hoc test (no Gaussian distribution). Intestinal segments in which the hypothetical expression value was not significantly different from zero were excluded from statistical analyses, what only applies to the SGLT1 expression in duodenal samples from both groups.

The approach for this statistical analysis was as following: If the means for the duodenal samples do not differ from zero while all other means are different from zero, then it can be concluded that the duodenum differs from the other intestinal segments. If the duodenum is included in the one‐way ANOVA, this leads to the result that the major effects are observed between the duodenum and the other intestinal segments but obvious differences between the other intestinal segments are not well reflected. Thus, we excluded the duodenum from the one‐way ANOVA in order to get a more precise analysis of the differences between the remaining intestinal segments. Statistical analyses were performed with GraphPad Prism 6.05 and significant values of *P *<* *0.05, *P *<* *0.01, *P *<* *0.001 are indicated.

## Results

### Starch and glucose contents

**Figure 2:**
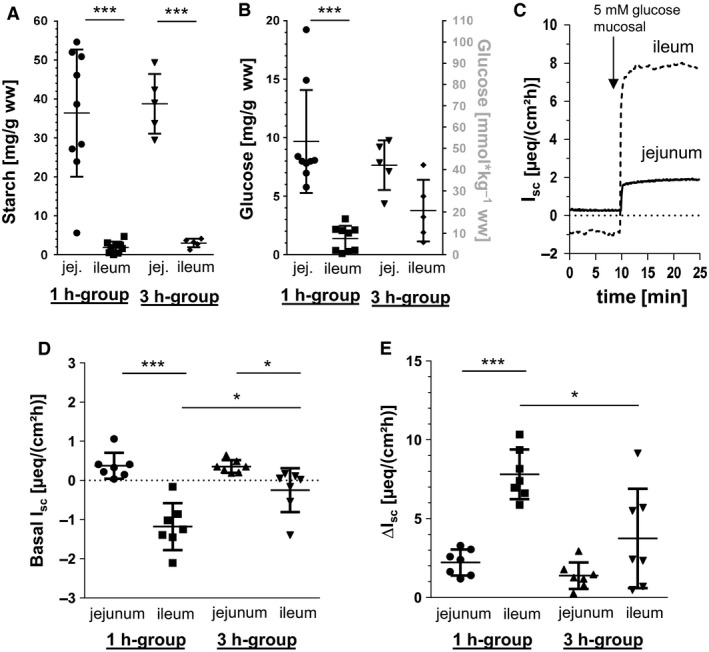
Luminal starch and glucose contents, basal *I*_sc_ as well as glucose‐induced changes in *I*_sc_ (∆*I*_sc_) in porcine jejunum and ileum. Starch (A) and glucose concentrations (B) in jejunal and ileal chyme taken from 1 h‐group (*N* = 9) and 3 h‐group (*N* = 5) in relation to chyme wet weight (ww). Scatter plots with means ± SD. (C) Time course (exemplarily from one animal) of jejunal and ileal *I*_sc_ after the addition of glucose (5 mmol L^‐1^ mucosal)), (D) Basal *I*_sc_ without mucosal glucose and (E) increases in *I*_sc_ after mucosal administration of 5 mmol L^−1^ glucose in Ussing chambers using jejunal and ileal tissue from pigs of 1 h‐group (*N* = 7) or 3 h‐group (*N* = 7). Scatter plots with means ± SD. Statistical analyses: Student's unpaired *t*‐test. Significant differences between columns are indicated as * (*P* < 0.05), ***(*P* < 0.001).

The jejunal starch contents in the 1 h‐group as well as in the 3 h‐group were >10fold higher than in the ileum (*P *<* *0.001) (Fig. 2A). While the jejunal glucose contents in the 1 h‐group were significantly higher compared with the ileum (*P *<* *0.001), the ileal glucose contents in the 3 h‐group increased up to values comparable with the jejunum (Fig. 2B).

### Basal and glucose‐induced *I*_sc_ and *G*_t_

The basal ileal *I*_sc_ of 1 h‐pigs were significantly more negative compared with the jejunum (*P *<* *0.001) (Fig. 2C) which could also be observed to a lesser extent in 3 h‐pigs (*P *<* *0.05). In these tissues, the basal ileal *I*_sc_ were significantly less negative compared with the respective ileal *I*_sc_ of the 1 h‐group (*P *<* *0.05). The increases in *I*_sc_ after mucosal administration of 5 mmol L^−1^ glucose were significantly higher in ileal tissues compared with jejunal tissues in the 1 h‐group (*P *<* *0.001) (Fig. 2D). However, in the 3 h‐group, the glucose‐induced increases were significantly lower in both intestinal segments compared with the 1 h‐group (*P *<* *0.05) resulting in a significantly different but less pronounced ∆*I*_sc_ in the ileum compared with the jejunum.

No significant correlations between glucose‐induced ∆*I*_sc_ and glucose content in chyme were found considering the single groups, but in the case of the 1 h‐group only three animals were available for this correlation. When combining data from both groups and intestinal locations, a significant negative correlation (*P *<* *0.05, *R* ^2 ^= 0.43) between ∆*I*_sc_ and chyme glucose content is found.

Basal *G*_t_ was not different between the groups or the intestinal segments (*G*_t basal_ (mS cm^−2^): 1 h‐group: jejunum 27.85 ± 8.53, ileum 25.88 ± 3.06; 3 h‐group: jejunum 22.50 ± 5.97, ileum 28.30 ± 6.88). Changes in *G*_t_ 10 min after the mucosal addition of glucose (time point at which Δ*I*_sc_ was assessed) were not significant (Δ*G*_t glucose_ (mS cm^−2^): 1 h‐group: jejunum −1.05 ± 2.16, ileum 1.60 ± 2.04; 3 h‐group: jejunum −1.07 ± 1.68, ileum 0.41 ± 2.06). No effects of Epi were observed (data not shown).

### mRNA expression‐copy number of RSP18

Copy numbers of RSP18 were stable along the intestinal axis in 1 h and 3 h‐pigs but copy numbers were slightly higher in the 3 h‐group (data not shown). The copy numbers were stable along the intestinal axis in both groups. However, copy numbers were slightly higher in the 3 h‐group. To account for this, normalized copy numbers for 1 h and 3 h‐pigs in one intestinal segment were never compared directly but only relative changes in the expression pattern are discussed.

### SGLT1 expression along the small intestinal axis

**Figure 3:**
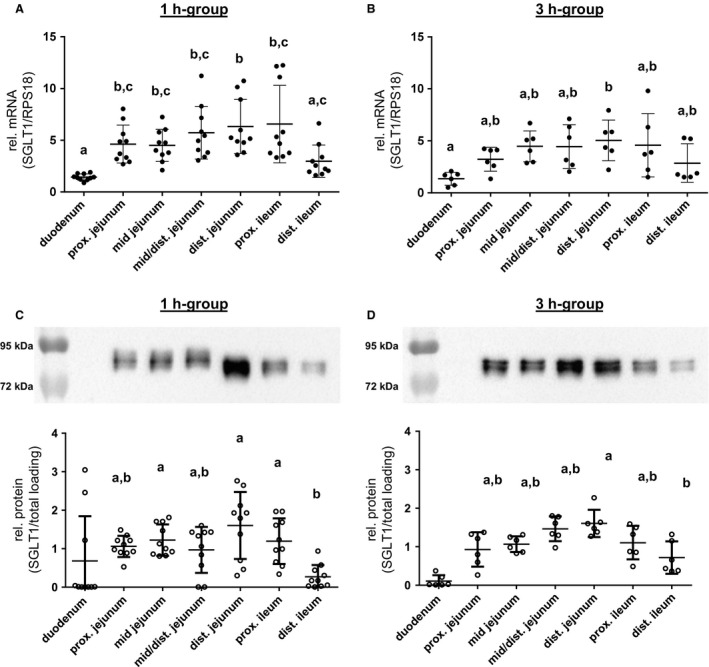
SGLT1 expression along the small intestinal axis. The SGLT1 mRNA expression levels detected in porcine small intestinal segments of (A) 1 h‐group (*N* = 10) and (B) 3 h‐group (*N* = 6) animals are shown. Copy numbers were normalized against RPS18 mRNA values. SGLT1 protein expression along the small intestines is presented relative to total protein loading, and representative western blots of the 1 h‐group (*N* = 10) (C) and 3 h‐group (*N* = 6) (D) are included. Scatter plots with means ± SD. Statistical analyses: ANOVA, Kruskal–Wallis test, Dunn's multiple comparisons test. Intestinal segments not sharing the same letters are significantly different (*P* < 0.05). The duodenal SGLT1 contents in both groups (C, D) were not different from the hypothetical value “0” and excluded from the ANOVA in order to get a more precise analysis of differences between the other intestinal segments, as also explained in the statistics section.

In the 1 h‐group, gene expression of SGLT1 was lowest in the duodenum compared with jejunal segments of the proximal (*P *<* *0.01), mid (*P *<* *0.01), mid/distal (*P *<* *0.001), or distal part (*P *<* *0.001), as well as the proximal ileum (*P *<* *0.001), and in the distal ileum compared with the distal jejunum (*P *<* *0.05), while the mRNA distribution along the jejunum and proximal ileum did not differ (Fig. 3A). Differences in SGLT1 gene expression were attenuated in the 3 h‐group but again the duodenum exhibited the lowest mRNA level (*P *<* *0.05, compared with distal jejunum) (Fig. 3B).

Detection of SGLT1 protein in the BBM resulted in a double band with an approximate molecular mass of about 80 kDa (Fig. 3C and D, upper part) which is higher than the calculated molecular weight of 72 kDa due to N‐glycosylation (Herrmann et al. 2012). Specificity of the SGLT1 antibody was verified using a control peptide (ab190911, Abcam) (data not shown).

In both groups, the statistical analyses revealed no significant SGLT1 abundance in the duodenum (Fig. 3C and D). The abundance of apical SGLT1 along the small intestines was similar from the proximal jejunum to the proximal ileum in both groups. SGLT1 contents in the distal ileum were significantly lower in the 1 h‐group compared with the mid jejunum (*P *<* *0.05), distal jejunum (*P *<* *0.001), or proximal ileum (*P *<* *0.05) and also in the 3 h‐group compared with the distal jejunum (*P *<* *0.05). Aside from these moderate changes of the mean expression along the intestinal axis, no significant relations were found when correlating mRNA and protein levels of SGLT1 (data not shown). Protein levels of SGLT1 (and also pSGLT1) were also not significantly correlated with changes in *I*_sc_ (data not shown), but as shown in Figure 1, the sampling sites for Ussing chamber experiments and expression analysis were not exactly the same, which might have led to a higher scattering and might have prevented a significant correlation.

The same applies to the correlation of SGLT1 expression and luminal glucose contents but between these parameters, a connection was found when analyzing all data together irrespective of intestinal location or group assignment, whereby only four animals could be analyzed in this context For SGLT1, there is a significant positive correlation (*P = *0.017, *R* ^2 ^= 0.344), while no significance was observed for pSGLT1/SGLT1 (*P* = 0.158, *R* ^2^ = 0.147).

### pSGLT1 (Ser418) abundance along the small intestinal axis

**Figure 4:**
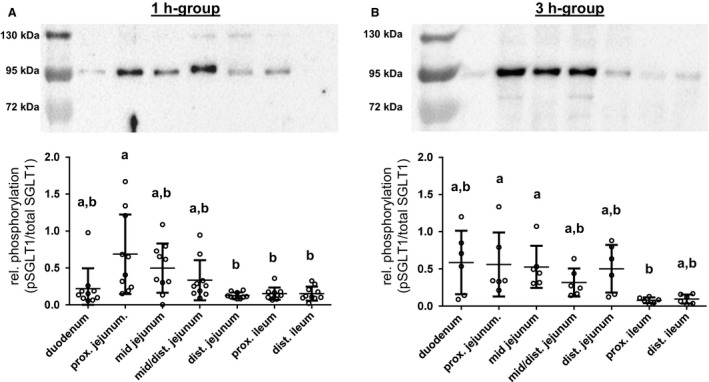
pSGLT1 (Ser418) abundance along the small intestinal axis in relation to total SGLT1 protein contents and representative western blots of pSGLT1 (Ser418) signals in the 1 h‐group (*N* = 10) (A) and 3 h‐group (*N* = 6) (B) are included. Scatter plots with means ± SD. Statistical analyses: Kruskal–Wallis test, Dunn's multiple comparisons test. Intestinal segments not sharing the same letters are significantly different (*P* < 0.05).

Detection of pSGLT1 using a custom‐made antibody (Ser418) in BBM resulted in a single band with a molecular mass of approximately 95 kDa (Fig. 4A and B). Phospho‐specificity was verified by dephosphorylation using lambda protein phosphatase (New England Biolabs GmbH, Frankfurt/Main, Germany) (data not shown).

Along the intestinal segments, the pSGLT1 (Ser418) contents in the 1 h‐group were similar, except for the proximal jejunum which revealed significantly higher phosphorylation compared with the distal jejunum (*P *<* *0.01) as well as the proximal (*P *<* *0.05) and distal ileum (*P *<* *0.05) (Fig. 4A). Significant differences in the 3 h‐group were found between the proximal ileum and the proximal jejunum (*P *<* *0.05) or mid jejunum (*P* < 0.05) (Fig. 4B). The relative phosphorylation in the distal jejunum increased significantly compared with the 1 h‐group (*P *<* *0.01).

We calculated the mean values of intestinal segments adjacent to the tissues which were used for Ussing chamber studies from each pig. The resulting data revealed higher mean jejunal phosphorylation of SGLT1 compared with the mean ileum values in the 1 h‐group (*P *< 0.01, *N* = 10) as well as in the 3 h‐group (*P *< 0.05, *N* = 6).

### GLUT2 expression along the small intestinal axis

**Figure 5:**
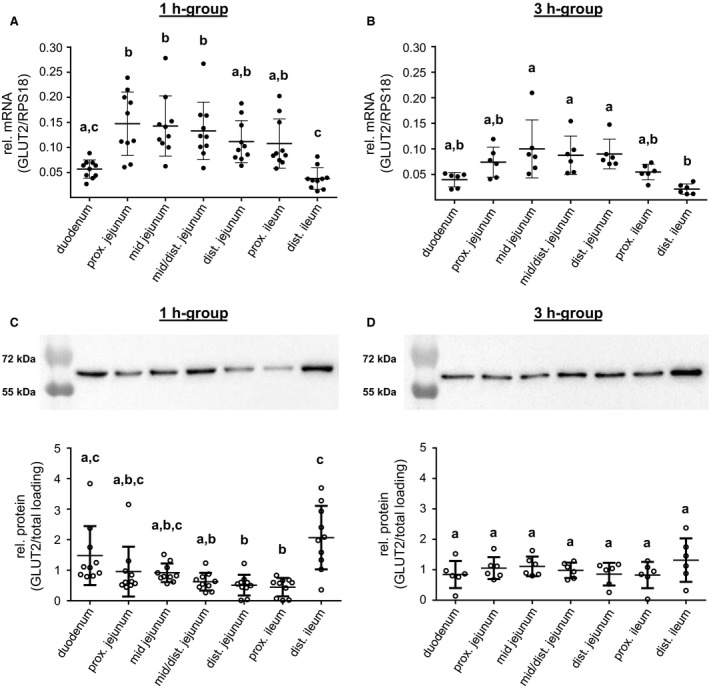
GLUT2 expression along the small intestinal axis. The GLUT2 mRNA expression levels detected in porcine small intestinal segments of (A) 1 h‐group (*N* = 10) and (B) 3 h‐group (*N* = 6) animals are shown. Copy numbers were normalized against RPS18 mRNA values. GLUT2 protein expression along the small intestines is presented relative to total protein loading and representative western blots of the 1 h‐group (*N* = 10) (C) and 3 h‐group (*N* = 6) (D) are included. Scatter plots with means ± SD. Statistical analyses: ANOVA, Kruskal–Wallis test, Dunn's multiple comparisons test. Intestinal segments not sharing the same letters are significantly different (*P* < 0.05).

Similar to SGLT1, the GLUT2 mRNA distribution in the 1 h‐group was lowest in duodenum compared with the proximal (*P *< 0.01), mid (*P *< 0.01), or mid/distal jejunum (*P *< 0.05) and in the distal ileum compared with the proximal (*P *< 0.001), mid (*P *< 0.001), mid/distal (*P *< 0.01), or distal jejunum (*P *< 0.05) as well as the proximal ileum (*P *<* *0.05) (Fig. 5A). Again, the differences were attenuated in the 3 h‐group leading to loss of significance of duodenal expression compared with the other segments. In the distal ileum, the GLUT2 mRNA abundance was significantly lower compared with the mid, mid/distal or distal jejunum (*P *<* *0.01, respectively) (Fig. 5B).

Detection of GLUT2 protein in CM resulted in a band with approximatly 60 kDa molecular mass which is slightly higher than the calculated molecular mass of 54 kDa (Fig. 5C and D). Proof of antibody specificity using the respective control peptide (ABIN939557, antibodies‐online) led to disappearance of the band (data not shown).

In 1 h‐pigs, the duodenum and distal ileum possessed significantly more GLUT2 proteins compared with the distal jejunum (*P *<* *0.01, respectively) or proximal ileum (*P *<* *0.01, *P *<* *0.001, respectively) (Fig. 5C). Furthermore, the GLUT2 content in the distal ileum was significantly higher than in the mid/distal jejunum (*P *<* *0.05) (Fig. 5C). GLUT2 abundance showed no substantial differences throughout the small intestinal axis in the 3 h‐group (Fig. 5D).

### Adrenergic *β*_2_‐receptor expression in the small intestinal mucosa

**Figure 6:**
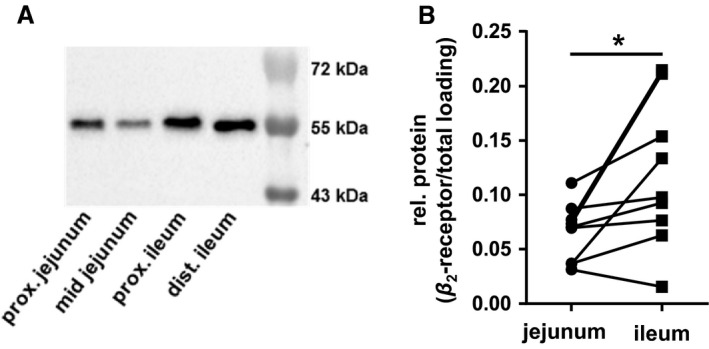
Adrenergic *β*_2_‐receptor expression in the small intestines of 1 h‐pigs. (A) Representative western blot showing adrenergic *β*_2_‐receptor expression in jejunum and ileum taken from areas surrounding the tissue samples used for Ussing chamber and incubation studies. (B) Relative amounts of adrenergic *β*_2_‐receptor in jejunum or ileum was estimated for each animal by determination of its abundance in relation to total protein loading in proximal and mid jejunum samples as well as the proximal and distal ileum and subsequent calculation of the respective mean value for each segment (*N* = 9). Statistical analyses: Student's unpaired *t*‐test. Significant differences between intestinal segments are indicated as * (*P* < 0.05).

As a prerequisite, for determining whether electrogenic responses to glucose or basal *I*_sc_ could be modified by Epi, we examined abundance of *β*_2_‐adrenoceptors in jejunal and ileal tissues from the 1 h‐group. Detection of the *β*_2_‐adrenoceptor resulted in a single band with an approximate molecular mass of 55 kDa which is slightly higher than its calculated molecular mass of 47 kDa. Proof of antibody specificity was not possible because a respective control peptide was not available. The band was detectable in both segments (Fig. 6A). Comparison of the mean abundances revealed significantly higher expression in the ileum compared with the jejunum (*P *<* *0.05, *N* = 9) (Fig. 6B).

### Effect of Epi on glucose‐induced and basal *I*_sc_

**Figure 7:**
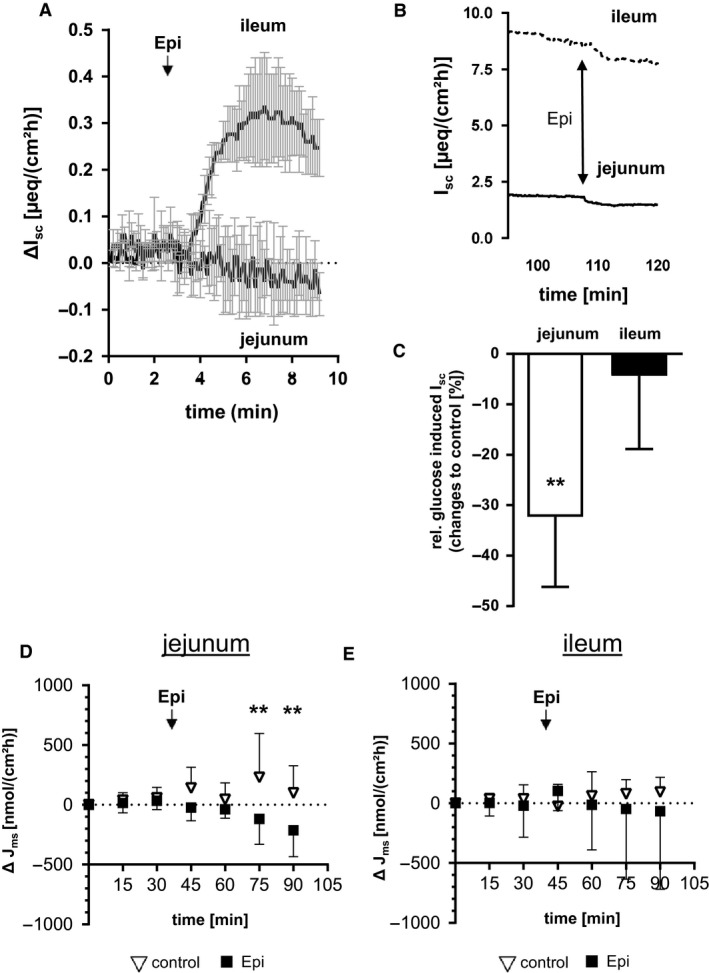
Effects of Epi on electrogenic transport features in mucosa of 1 h‐pigs. (A) Changes in basal *I*_sc_ (*μ*eq∙cm^−2^ h^−1^) after serosal administration of 50 *μ*mol L^−1^ Epi in Ussing chambers using jejunal and ileal mucosa (*N* = 3, means ± SD). The time courses of Δ*I*_sc_ during 9 min are shown for each intestinal segment (Epi administration is indicated by arrows). (B) time course (exemplarily from one animal) of already glucose‐stimulated jejunal and ileal *I*_sc_ after the addition of 50 *μ*mol L^−1^ Epi (C) Changes in glucose‐induced *I*_sc_ (*μ*eq cm^−2^ h^−1^) 15 min after serosal administration of 50 *μ*mol L^−1^ Epi compared with the respective control in Ussing chambers (*N* = 5). Significant differences between Epi and solvent control were analyzed by Student's unpaired *t*‐test and indicated as ** (*P* < 0.01). Means ± SD. (D, E) Epi‐induced effects on glucose mucosal‐to‐serosal flux rates (J_ms_) in the porcine jejunum. Changes of *J*_ms_ (Δ*J*_ms_ (nmol cm^−2^ h^−1^)) for control chambers (triangles) and Epi‐treated chambers (squares) for jejunal (D) or ileal (E) mucosa are shown (*N* = 5, means ± SD). Statistical analyses were performed using multiple *t*‐test. Significant differences are indicated as ** (*P* < 0.01), *** (*P* < 0.001).

The jejunal glucose‐induced *I*_sc_ declined within 15 min by 32% (*P *<* *0.01), while the ileal *I*_sc_ remained unaffected (Fig. 7B). In contrast to changes in glucose‐induced *I*_sc_, Epi increased the basal *I*_sc_ in the ileum but not in the jejunum (Fig. 6A). Modulations of glucose‐induced *I*_sc_ by previous Epi administration could not be observed. Addition of *β*‐adrenoceptor inhibitor propranolol or PKA inhibitor H‐89 could not prevent the effects of Epi before or after glucose administration (data not shown).

### Effect of Epi on glucose *J*_ms_

As the *I*_sc_ is a measure of net transepithelial ion transfer, it may not quantitatively reflect the ion transfer solely caused by Na^+^/glucose co‐transport since Na^+^ or glucose influxes may also influence other ion movements across the intestinal epithelium. Therefore, we also determined mucosal‐to‐serosal glucose flux rates (*J*_ms_) before and after the administration of Epi. No effects of Epi were obvious considering the means before and after treatment with solvent or Epi (Jejunum: 847 ± 313 to 1078 ± 517 *μ*mol cm^−2^ h^−1^ for control chambers vs. 731 ± 277 to 518 ± 238 *μ*mol cm^−2^ h^−1^ for epi chambers, Ileum 1488 ± 755 to 1542 ± 709 *μ*mol·cm^−2^ h^−1^ for control chambers vs. 1723 ± 1495 to 1658 ± 1057 *μ*mol·cm^−2^ h^−1^ for epi chambers). This was due to the high initial variation between the individual chambers in comparison to the Epi effects. Significant effects were observed regarding the changes in J_ms_ when comparing data at 0 min to data at 75 or 90 min (ΔJ_ms_). As shown in Figure 7C, jejunal Δ*J*_ms_ decreased within 30 min after Epi treatment compared to control chambers (Fig. 7C), while no changes were observed for ileal samples (Fig. 7D).

### Epi effects on activity of protein kinases

**Figure 8:**
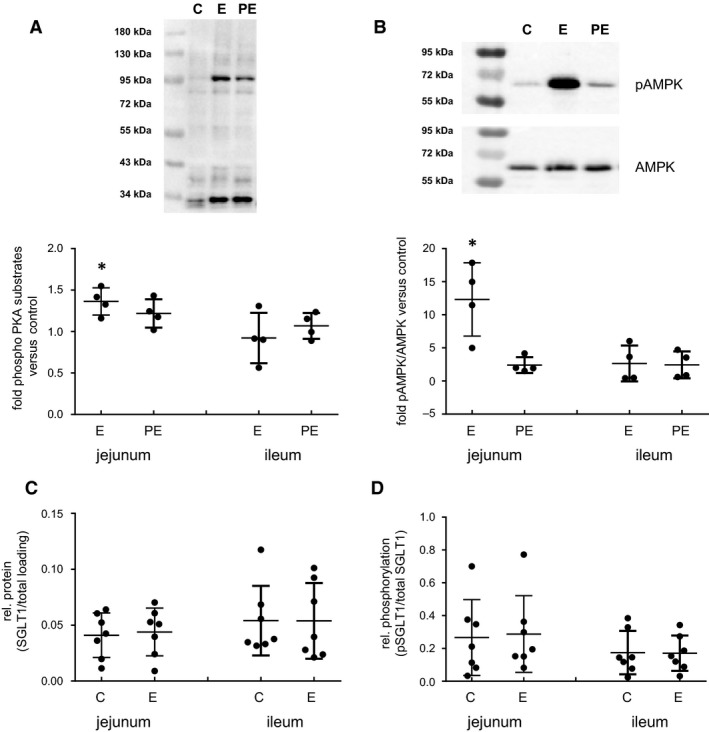
Effects of Epinephrine (Epi) on the amount of phosphorylated PKA substrates, AMPK phosphorylation, SGLT1, and pSGLT1 (Ser418) abundance in stripped mucosa of 1 h‐pigs. (A) Amount of phosphorylated PKA substrates (B) relative pAMPK (Thr 172) contents in incubated stripped mucosa of porcine jejunum or ileum were determined by western blotting. Mucosa was incubated for 15 min with solvent control (=C) or 50 *μ*mol L^−1^ Epi (=E). In parallel, 20 *μ*mol L^−1^ propranolol were added 20 min prior to Epi administration (=PE). Results are presented in relation to control (set as 1) as scatter plots with means ± SD (*N* = 4) and representative western blots done with jejunal samples are included. Apical abundance of (C) SGLT1 or (D) pSGLT1 (Ser418) relative to total SGLT1 protein in stripped mucosa of porcine jejunum or ileum after 15 min incubation with solvent control (=C) or 50 *μ*mol L^−1^ Epi (=E). Scatter plots with means ± SD (*N* = 7). Statistical analyses: Student's unpaired *t*‐test. Significant differences to control are indicated as * (*P* < 0.05).

As *β*_2_ receptor binding by Epi is known to activate PKA, it was necessary to check if this PKA activation by Epi treatment occurred under the applied experimental conditions. No differences in the PKA activity were detectable using the PepTag^®^ Assay. In an alternative approach, we indirectly estimated changes in PKA activity by western blotting assays performed with cytosol preparations of Epi and propranolol‐treated tissues using an antibody directed against the phosphorylated PKA consensus motif for nonspecific detection of PKA phosphorylated proteins. Lysates taken from Epi‐treated jejunal mucosa possessed significantly more phosphorylated PKA substrate than the control (*P *<* *0.05) (Fig. 8A), whereas no differences were observed in the ileum. Preincubation with propranolol resulted in disappearance of the Epi effect. Downstream PKA targets were also influenced by Epi. Similar to PKA activity the phosphorylation of AMPK at Thr172 was significantly higher in jejunal Epi‐treated samples compared with the solvent control (*P *<* *0.05) (Fig. 8B). No effect could be observed in ileal samples. Inhibition of *β*‐adrenoceptors by propranolol prevented Epi‐induced increases in pAMPK (Thr172) in the jejunum.

### Effect of Epi on SGLT1 and pSGLT1 abundance

The Epi‐incubated stripped mucosa from jejunal and ileal tissues of the 1 h‐group were also used for analyzing pSGLT1 levels. In both, jejunum and ileum, no changes in the total SGLT1 abundance could be observed after administering Epi (Fig. 8C). Also, the phosphorylation level of SGLT1 at Ser418 remained unaffected after Epi treatment in both segments (Fig. 8D).

## Discussion

In contrast to mice(Yoshikawa et al. 2011) or horses(Dyer et al. 2009), in the 1 h‐pigs, the abundance of SGLT1 mRNA was lowest in the duodenum and, whereas murine SGLT1 abundance decreases along the small intestinal axis, in the porcine small intestines, SGLT1 presence was similar throughout the whole jejunum and the proximal ileum. Only the distal ileum, possessed markedly lower SGLT1. The relative distribution of apical SGLT1 protein in the small intestinal segments was in accordance with the respective mRNA levels.

Differences to mice were also obvious regarding the distribution of GLUT2 mRNA. In mice, the mRNA abundance decreased from the proximal to distal small intestines (Yoshikawa et al. 2011), whereas porcine GLUT2 gene expression in the 1 h‐group was lowest in the duodenum and distal ileum but not different between the segments in between. Interestingly, GLUT2 protein abundance differed in the duodenum and distal ileum from mRNA levels. Such discrepancy between mRNA and protein levels was also found for SGLT1, GLUT2, and GLUT5 in mouse small intestines (Fatima et al. 2009). The low SGLT1 abundance in these segments may indicate a minor role of the duodenum and the distal ileum in glucose absorption and for adequate energy supply of the intestinal epithelium, glucose is mainly transported from the blood into enterocytes by basolateral GLUT2. Indeed, we could find a negative correlation between the protein expression levels of GLUT2 and SGLT1 when analyzing all data together irrespective of feeding time or intestinal segment (*P = *0.0057, *R* ^2^ = 0.107, *y* = −0.31∙*x* + 1.34, *n* = 70). It can only be speculated regarding the discrepancy between relative GLUT2 mRNA and protein levels especially in the distal ileum. Conceivable mechanisms might be related to segment‐specific elongation of mRNA half‐life or differences in GLUT2 protein turnover.

The advancing digestion process in the 3 h‐group resulted in increased luminal glucose contents in the ileum that no longer differed from jejunal glucose contents as was the case in the 1 h‐group. Differences in SGLT1 and GLUT2 expression at both, mRNA and protein levels, were more moderate between segments in the distal small intestine compared to the 1 h‐group but showed similar distributions overall. Tissue from the 3 h‐group showed altered electrophysiological properties in Ussing chamber experiments. The basal *I*_sc_ was more positive in the ileum. The electrogenic response to glucose was slightly decreased in the jejunum and, more pronounced, in the ileum of the 3 h‐group compared with the 1 h‐group although higher glucose contents were measured in the ileal chyme of 3 h animals.

It seems that short‐term reception of luminal glucose modulates protein abundance and even gene expression of SGLT1 or GLUT2 at a moderate level. It has already been reported that SGLT1 expression is enhanced at high carbohydrate diets, thus, these observations were made under long‐term dietary conditions in weaned piglets (Moran et al. 2010) or horses (Dyer et al. 2009) and diet‐induced changes in histone acetylation levels in the SGLT1 gene could be detected in mice (Honma et al. 2009). In contrast to reduced electrogenic glucose absorption in the short‐term situation, the long‐term studies described an adaptive increase in glucose uptake in the proximal and mid small intestines in weaned piglets (Moran et al. 2010) or in the duodenum, jejunum, and ileum in horses (Dyer et al. 2009). When interpreting the results regarding the 3 h‐group, it should definitely to be taken into account, that the *I*_sc_ was measured. One has also to consider glucose may induce changes in other ion currents then Na^+^. For example, SGLT1 activity may induce Ca^2+^ influx (Kellett 2011), and it has been shown, that glucose is able to induce Ca^2+^‐dependent Cl^−^ secretion (Yin et al. 2017), what may be part of the observed effects.

Nevertheless, as a potential regulatory mechanism we found evidence for differences in SGLT1 phosphorylation at Ser418 which has been described to mediate conformational changes of the transporter (Subramanian et al. 2009). The average relative SGLT1 phosphorylation levels of segments surrounding the functionally examined mucosal tissues were higher in the jejunum compared with the ileum. Consequently, the combination of functional and phosphorylation data suggest that Ser418 phosphorylation in the porcine system may not increase but in fact decrease SGLT1 activity. A decrease in the uptake of alpha methyl‐D‐glucopyranoside (AMG) has also been found in primary renal proximal tubule cells after 24 h Epi treatment (Kim et al. 2007). Nevertheless, the majority of related studies reported increases in SGLT1‐mediated transport of glucose or glucose analogues after Epi treatment or PKA stimulation (Hirsch et al. 1996; Ishikawa et al. 1997; Aschenbach et al. 2002; Louzao et al. 2003; Subramanian et al. 2009).

For further investigation of the conflicting observations, we stimulated jejunal and ileal stripped mucosa with Epi to induce PKA‐mediated phosphorylation of SGLT1 at Ser418 *via* the β_2_‐adrenergic receptor (Ishikawa et al. 1997; Aschenbach et al. 2002). Surprisingly, both, the total SGLT1 abundance in BBM as well as its relative phosphorylation were not influenced by Epi. However, functional proof of Epi effects in Ussing chambers revealed that the electrogenic response to glucose and even glucose m/s‐flux rates decreased in the jejunum. This functional effect is in agreement with increased amounts of phosphorylated PKA substrates and AMPK phosphorylation only found in jejunal but not ileal tissue samples. The fact that Epi treatment resulted segment specifically in PKA activation and subsequently in decreased glucose transport without affecting pSGLT1 (Ser418) levels points to a transporter‐independent mechanism which is supported by Epi‐induced rapid increases in basal *I*_sc_ without mucosal glucose only in the ileum.

As PKA action is not restricted to glucose transport but reflects a central intracellular regulatory tool with regard to several other signaling pathways including modulation of several ion transporters, it might be suggestible that the decreases in electrogenic glucose transport are secondary effects induced by changes of the apical membrane potential (Kimmich and Randles 1988). Depolarization of the apical membrane caused by Na^+^‐coupled transport itself is balanced, for example, by apical or basolateral K^+^ secretion (Heitzmann and Warth 2008) since a continuous depolarization of the apical membrane would result in diminished driving forces for Na^+^/glucose co‐transport and most likely to a decreasd SGLT1 activity (Umbach et al. 1990). Additionally, other Na^+^‐driven apical transporters such as Na^+^/H^+^ exchangers (NHE) might interfere with SGLT1 activity (Coon et al. 2011). A study on effects of Epi on colonic ion transport in rats demonstrated modifications of K^+^ and Cl^−^ transepithelial flux rates in a segment‐specific manner (Horger et al. 1998) which was also shown for electrogenic K^+^ secretion in the guinea pig colon after activation of *β*_1_‐ and *β*_2_‐adrenoceptors (Zhang et al. 2009).

Differences in responsiveness to Epi between the porcine jejunum and ileum may be based on the fact that these intestinal segments possess unique transporter and ion channel settings reflecting the demands depending on different local conditions generated by digestive processes, for example, luminal pH or glucose availability. The general influence of luminal pH on membrane trafficking of ion transporters, for example, CFTR or NHE3, has been shown using rat duodenal, jejunal, and ileal intestinal loops (Jakab et al. 2012). Nonetheless, also the receptor equipment appears to be segment‐specific because the *β*‐adrenoceptor inhibitor propranolol could not inhibit Epi‐induced decreases in jejunal glucose transport or ileal increase in basal *I*_sc_ as functional read‐outs but reduced Epi‐mediated increases in the amount of phosphorylated PKA substrates or AMPK phosphorylation in the jejunum. We could detect the *β*_2_‐adrenergic receptor in both segments but the very restricted inhibitory potential of propranolol points to alternative Epi binding receptors. It has been shown that small intestinal enterocytes may also possess functional *α*_1_‐ and *α*_2_‐adrenoceptors (Chang et al. 1983; Baglole et al. 2006). Activation of *α*_1_‐adrenoceptors in rat small intestinal enterocytes led to decreased chloride secretion by downregulation of the calcium‐activated chloride channel ClC‐2 (Baglole et al. 2006, 2007). Other potential explanations for the observed Epi effects as *α*_1_‐adrenoceptor‐mediated activation of protein kinase C *via* PLC/IP3/DAG pathway and subsequent downregulation of CFTR (Shen et al. 1993) were not addressed in this study. Besides a downregulation of CFTR by PKC, it has also been shown, that CFTR phosphorylation by PKC is to some extent required for a PKA‐mediated activation (Jia et al. 1997). Additionally, it could not be excluded, that Epi may bind to dopamine receptors at the used concentration. Epi binding has been shown for the D4 receptor (Lanau et al. 1997) and the D1 receptor has been found, for example, in rat enterocytes in the whole small intestines (Vaughan et al. 2000). Since the activation of D1 receptor stimulates the activity of adenylate cyclase, Epi binding to the D1 receptor may explain why the activation of PKA in this study was insensitive to propranolol.

The complex issue of Epi effects (and the respective receptors) on intestinal ion transport should be elucidated in further studies. Altogether, it is well conceivable that differences in transporter settings, its respective temporary apical abundance and/or the responsiveness to PKA stimulation as well as environmental differences in pH or glucose availability might explain the basal and Epi‐induced segment‐specific features in our study.

This consideration is strengthened by verification of the observation that the basal *I*_sc_ in the porcine jejunum was unlike the ileum (Herrmann et al. 2012) or by the recently published observation that phlorizin binding to BBM vesicles taken from the porcine jejunum was less Na^+^‐specific compared with the ileum (Herrmann et al. 2016). Unique ion flux conditions would affect the electrogenic glucose transport features mainly by modifying the apical membrane potential, resulting in stimulation or attenuation of SGLT1 activity by hyperpolarization or depolarization, respectively. Under the assumption that phosphorylation of SGLT1 at Ser418 increases its activity, enhanced pSGLT1 (Ser418) levels may compensate for depolarizing factors which might naturally occur in the jejunum compared with the ileum or appear during the proceeding digestion as well as after PKA activation induced by Epi treatment. Although we observed lower glucose absorption connected with more phosphorylated SGLT1 in the jejunum, we are unable to estimate the potential decrease in intestinal glucose transport without phosphorylation which might be even more pronounced under depolarizing conditions.

Summarizing the present data with regard to the three aims of this study, it can be stated that the distribution of SGLT1 and GLUT2 along the porcine small intestinal axis on mRNA and protein levels in relation to proceeding of digestive processes has been demonstrated for the first time. However, none of these measures explains the functional differences between jejunum and ileum.

The relative amount of phosphorylated SGLT1 (Ser418), could be quantified for the first time in porcine tissue samples along the intestinal axis instead of cell culture by use of a new antibody. Although pSGLT1 (Ser418) reflects a putatively more active transporter, its relative abundance was even higher in the less active jejunum.

Epi incubation activated PKA as intended but the segment‐specific effects on basal *I*_sc_ and glucose transport were in part surprising and difficult to interpret. It has to be taken into account for further experiments, that not only PKA but also other pathways may be activated by Epi which may interact directly or indirectly with glucose transport. SGLT1 phosphorylation level at serine 418 did change with respect to the intestinal location and the luminal provision of glucose but not in response to Epi treatment. Whether increased phosphorylation of SGLT1 at PKA target Ser418 actually mediates the observed decreases in jejunal glucose absorption in pigs or if it represents a compensatory response to apical membrane depolarization has to be investigated in more detail in further studies.

## Conflict of Interest

The authors have no conflict of interest to declare.
